# Nigericin‐Triggered Phosphodynamics in Inflammasome Formation and Pyroptosis

**DOI:** 10.1002/pmic.70030

**Published:** 2025-09-02

**Authors:** Vanya Bhushan, Clinton J. Bradfield, Sandhini Saha, Sung Hwan Yoon, Iain D. C. Fraser, Aleksandra Nita‐Lazar

**Affiliations:** ^1^ Functional Cellular Networks Section Laboratory of Immune System Biology, NIAID Bethesda Maryland USA; ^2^ Signaling Systems Section, Laboratory of Immune System Biology, NIAID Bethesda Maryland USA

**Keywords:** inflammasome, inflammation, MAPK, phosphoproteomics, pyroptosis

## Abstract

Innate immune signaling relies heavily on phosphorylation cascades to mount effective immune responses. Although traditional innate immune signaling cascades following TLR4 stimulation have been investigated through a temporally quantitative phosphoproteomic lens, far fewer studies have applied these methods to distinct signaling following the inflammasome trigger leading to IL‐1β release. Here, we conducted time‐resolved phosphoproteomic profiling to investigate kinase signaling downstream of the inflammasome trigger nigericin. We found that nigericin induces rapid and potent alterations in the phosphorylation landscape where immune‐related signaling, mitogen‐activated protein kinases (MAPKs), and PKC signaling are prevalent. We also found significant evidence of phospho‐modified metabolic cascades, suggesting that phosphosignaling plays a role in previously described immunometabolic regulation. These signaling events preceded robust phosphorylation of DNA damage and chromatin reorganization proteins before pyroptotic rupture. Lastly, by performing temporal clustering of phospho‐dynamics, we revealed novel ontology‐level shifts in phosphosignaling cascades following nigericin treatment that highlight abrupt changes in cellular behavior during early and late intracellular inflammatory events.

**Summary:**

- Protein phosphorylation is critical to convey innate immune signaling information to specific effector arms of the cellular immune response.
- This study focuses on characterizing phosphoproteomic alterations stemming from the inflammasome trigger nigericin.
- By gaining a deeper understanding of global kinase phosphodynamics in response to inflammasome activation, we aim to identify novel pharmacological targets to treat chronic inflammatory diseases driven by inflammasome‐dependent IL‐1β release.

## 1 Introduction

Reversible protein phosphorylation facilitates rapid and dynamic biochemical regulation of cellular catalytic hubs in response to homeostatic imbalance. Over the course of evolution, hierarchies of intermingled kinases and phosphatases have matured to mediate specific changes in cellular behaviors. This signaling infrastructure bolsters many sensory responses and refines biochemical outputs through layered amplification and cross‐regulation [1, 2, 3]. Although phosphorylation is thought to directly modify between 30% and 75% of all cellular proteins at a given time [4, 5], recent advances in mass spectrometry sensitivity and throughput can now be applied to more broadly reveal omics‐level phosphorylation fluxes associated with induced cellular responses.

A prime example of this can be seen in receptor stimulation studies, such as toll‐like receptor (TLR) signaling, following exposure to pathogens. Here, phosphoproteomics has illuminated how numerous parallel signaling pathways integrate into core kinase nodes associated with Myd88 and Trif [6, 7, 8, 9, 10]. These core scaffolds facilitate Irak:Tab, Nemo:Tbk1, and mitogen‐activated protein kinase (MAPK) cascades to govern activation of NFkB, Irf3, and AP1 transcription factors that mediate extensive cytokine transcription [11]. In addition to these classical nodes, a tangential Syk:PKC signaling axis contributes to Nfat transcription factor activation, which has been highlighted for its role in establishing epigenetic imprinting on gene loci during innate immune training [12]. Similarly, immunometabolic regulation and activation of the transcription factor HIF‐1α have been linked to parallel PRKA:ERK signaling [13]. Although TLR‐driven phosphodynamics are well studied, the role of phosphoregulatory cascades triggered by inflammasome activators is incomplete.

Canonical inflammasome activation is governed by a two‐signal system where TLR or inflammatory cytokine receptor stimulation promotes an initial transcriptional priming signal, while a second signal acts as an intracellular stress trigger to activate sensory NLR proteins that seed the ASC inflammasome scaffold, leading to caspase activation and release of proinflammatory IL‐1 family cytokines [14, 15]. Although commonly used in vitro inflammasome activation protocols emphasize robust IL‐1β release [16], we have developed a time‐extended protocol to better separate the regulatory cascades governing the prime and trigger steps [17].

Recent studies aimed at monitoring phosphorylation of specific inflammasome sensory and effector components have suggested significant phospho‐regulation within the core canonical inflammasome scaffold components NLRP3 and ASC [18, 19, 20, 21, 22, 23]. These scaffolds harbor both positive and negative regulatory phosphorylation sites, which are regulated by various kinase systems. This highlights the need for distinct licensing steps that refine both inflammasome formation as well as downstream activity [15, 24]. Although these efforts have specifically aimed to identify phosphoregulation and other post‐translational modifications within core inflammasome components, understanding broader phosphoproteome dynamics has the potential to identify parallel regulatory elements that impinge upon inflammatory progression. These parallel refinement nodes could provide novel means to regulate distinct functions of inflammasome activity.

Various systems that govern parallel cellular behaviors must appropriately respond to specific cues within particular contexts. This central tenet of integrated cellular responses is exemplified in MAPK signaling. Here, diverse sensory inputs funnel into MAP4K:MAP3K:MAP2K:MAPK cascades into shared effector response nodes at the level of AP1 transcription factors. In addition to this integration response, each member kinase influences a specific set of other cellular proteins, allowing wide‐ranging specialization, particularly in immune and stress responses. Recent studies, including our own, have highlighted distinct roles for specific members of MAPK signaling families following inflammasome activation, revealing distinct temporal patterns of MAPK activation that mirrored the timing of inflammasome formation and the delayed onset of cellular pyroptosis [17].

Here, we assessed phosphoproteome dynamics associated with the inflammasome trigger event, aiming to create a more detailed map of the phosphosignaling initiated during this important inflammatory process. This information could be valuable for targeting specific aspects of the cellular inflammatory response.

## 2 Materials and Methods

### 2.1 Cells and Culture Conditions

U937 human myeloid leukemia cells (ATCC CRL1593.2) were cultured in 15 cm suspension plates to a final density of 2 × 10^6^ cells/mL in 5% FBS (HiClone) RPMI‐1640 (Gibco). Medium was refreshed, and PMA (Sigma) was added at a concentration of 20 ng/mL to allow macrophage differentiation for 2 days in 6‐well tissue culture plates. 6 × 10^6^ cells in three wells of a 6‐well plate were pooled for each sample prepared for each of a total of four independent replicates. Controls were also prepared to account for underlying sample drift. Fresh medium was added to samples treated with 200 nM KLA (Avanti polar lipids) for 18 h and their untreated counterparts. A final concentration of 10 µM of the inflammasome trigger nigericin was added directly to wells for the times indicated to assess additional trigger‐induced changes. Cells were lifted from wells and were washed in ice‐cold PBS, followed by immediate resuspension and lysis in 200 µL of room‐temperature 8 M urea (Sigma), 50 mM ammonium bicarbonate (ABC) (Sigma) buffer containing 2× Pierce protease inhibitor cocktail (Pierce) and 2× Phos‐stop (Roche). Lysates were sonicated briefly for 10 cycles (30 s on and 30 s off at 40°C) and clarified by centrifugation at 16,000 × *g* for 15 min at 4°C. Protein concentration was determined using the BCA assay (Thermo Scientific).

### 2.2 Protein Digestion for Global and Phosphoproteome Analysis

1 mg of each protein sample was reduced with 10 mM TCEP (tris(2‐carboxyethyl)phosphine) at 37°C for 45 min and alkylated with 20 mM CAM (chloroacetamide) at room temperature in the dark for 30 min. Samples were diluted eightfold with 50 mM ABC to reduce urea concentration below 1 M and digested overnight at 37°C with sequencing‐grade modified trypsin (Promega) at a 1:25 enzyme‐to‐substrate ratio for 18 h. For global proteome analysis, 50 µg of digested peptides per sample were desalted using C18 ZipTips (Millipore), vacuum dried, and reconstituted in 0.1% FA. 1 µg of desalted peptides was injected for LC‐MS/MS analysis. For phosphoproteome analysis, ∼1 mg of digested peptides per sample were subjected to phosphopeptide enrichment using the High‐Select TiO2 phosphoenrichment kit (Thermo) and High‐select Fe‐NTA Phosphopeptide Enrichment Kit (Thermo), following the manufacturer's protocols. Peptides were first incubated with a TiO_2_ column to selectively enrich di‐ and tri‐phosphorylated peptides. The flowthrough and wash fractions from the TiO_2_ step, which were expected to contain mono‐phosphorylated peptides, were subsequently subjected to enrichment using Fe‐NTA column.

To ensure comprehensive phosphopeptide coverage and maintain sample consistency, eluates from both TiO_2_ and Fe‐NTA enrichments were collected into the same tube, thereby combining the enriched fractions. The pooled eluates were immediately vacuum‐dried and reconstituted in 0.1% formic acid for LC‐MS/MS analysis. The enriched peptides were not desalted further (according to the manufacturer's protocol) and were directly injected into LC‐MS.

### 2.3 LC‐MS/MS Acquisition for Global and Phosphoproteome Samples

Mass spectrometry analysis for both global and phosphopeptide‐enriched samples was performed using a Thermo Orbitrap Fusion Eclipse (Thermo Fisher Scientific, San Jose, USA) coupled to a Thermo UltiMate 3000 (Thermo Fisher Scientific). For each run, 1 µg of total peptides was injected for LC‐MS/MS analysis. Peptides were first trapped on an Acclaim C18 PepMap 100 trap column (5 µm particles, 100 Å pores, 300 µm i.d. × 5 mm, Thermo Fisher Scientific) and then separated on a PepMap RSLC C18 column (2 µm particles, 100 Å pores, 75 µm i.d. × 50 cm, Thermo Fisher Scientific) at 40°C. The LC steps were: 98% mobile phase A (0.1% v/v formic acid in H2O) and 2% mobile phase B (0.1% v/v formic acid in ACN) from 0 to 5 min, 2%–35% linear gradient of mobile phase B from 5 to 155 min, 35%–85% linear gradient of mobile phase B from 155 to 157 min, 85% mobile phase B from 157 to 170 min, 85%–2% linear gradient of mobile phase B from 170 to at 172 min, 2% of mobile phase B from 172 to 190 min. Eluted peptides were ionized in positive ion polarity at a 2.1 kV of spraying voltage. MS1 full scans were recorded in the range of m/z 375 to 1500 with a resolution of 120,000 at 200 m/z using the Orbitrap mass analyzer. Automatic gain control and maximum injection time were set to standard and auto, respectively. The top 3 s data‐dependent acquisition mode was used to maximize the number of MS2 spectra from each duty cycle. Higher‐energy collision‐induced dissociation (HCD) was used to fragment selected precursor ions with a normalized collision energy of 27. MS2 scans were recorded using an automatic scan range with a resolution of 15,000 at 200 m/z using the Orbitrap mass analyzer.

### 2.4 Data Processing, Filtering, and Imputation for Global and Phosphoproteome Datasets

Raw files from global and phosphopeptide‐enriched samples were processed using Proteome Discoverer (PD 3.1) and searched using data‐dependent acquisition (DDA) against the Homo sapiens (taxon ID 9606). Identifications were filtered to retain only high‐confidence proteins and peptides, removing common contaminants and entries with fewer than two unique peptides. For phosphoproteome analysis, peptide‐level data containing phosphorylation site localization was used.

All analyses were performed using normalized intensity values exported from Proteome Discoverer. A missingness analysis was performed to assess data completeness across replicates by calculating the number of valid (non‐missing) values across four biological replicates (i.e., 0/4, 1/4, 2/4, 3/4, 4/4). The downstream analysis excluded proteins or peptides with values present in 0/4 or 1/4 replicates across all conditions. However, entries with 0/4 replicates in one condition and 4/4 replicates in another were retained to preserve condition‐specific signals. Only entries with at least 2/4 valid replicates in at least one condition were included for analysis.

Before imputation, the distribution of log2‐transformed intensities was visualized using Gaussian density plots to confirm approximate normality (Figure S1). Missing value imputation was performed separately for the two datasets: K‐nearest neighbor (KNN) imputation was applied to global proteome data, and mean/median‐based imputation was applied to phosphor‐proteome data set. Post‐imputation, Gaussian density plots were re‐generated to confirm that the imputation process did not introduce significant distributional shifts.

For downstream analysis, PD‐normalized intensities were first normalized to the 0 min untreated control (U937) for both nigericin and KLA conditions separately, and abundance ratios were then calculated by dividing nigericin by KLA values (N/K) at each corresponding time point (15, 30, 60, 120, and 180 min).  All data preprocessing, filtering, missingness analysis, and imputation were performed using R (version 4.3.1). Final processed and imputed datasets were saved as normalized input matrices for downstream statistical and pathway analyses. R packages and versions used are listed in Table S1.

### 2.5 Pathway Enrichment Analysis Using WebGestalt

Pathway enrichment analysis was performed using the WebGestalt (WEB‐based Gene SeT AnaLysis Toolkit) platform to identify biologically relevant pathways among differentially expressed proteins [25]. Global proteins were filtered using a log_2_ fold change (log_2_FC) threshold of ≥1 or ≤−1 for upregulated and downregulated proteins, respectively, while phosphoproteins were filtered based on a log_2_FC threshold of >0.58 for upregulation and <−0.58 for downregulation. The list of differentially expressed proteins, including their gene symbols, was submitted to WebGestalt for over‐representation analysis (ORA). The functional database for pathway analysis was set at Reactome.  The enrichment results were visualized as bar plots based on normalized enrichment scores (NES), and pathways with a false discovery rate (FDR) <0.05 were considered statistically significant. The figures were color‐coded according to FDR values and exported directly from WebGestalt for interpretation.  Further, the WebGestalt platform was used to predict kinase targets for identified candidate molecules.

### 2.6 Fuzzy Clustering of Temporal Profiles

To identify temporal expression patterns, fuzzy c‐means clustering was applied to the intensity profiles of global and phosphoproteins. For this analysis, only protein‐level data were considered. The extracted fold change from Nigericin/KLA (N/K) treatment time points at 15, 30, 60, 120, and 180 min—for clustering purposes was used. Proteins were grouped into 10 clusters based on their temporal expression patterns using fuzzy clustering, which allows each protein to belong to multiple clusters with varying degrees of membership. This method accounts for inherent biological variability and the gradual transitions in protein behavior over time. Clustering was conducted using standard fuzzy c‐means algorithms, and the resulting cluster assignments were used to create line plots and heatmaps that illustrate the dynamics of each cluster over time. Clusters exhibiting distinct trends were visualized individually, and all clusters were also combined to assess global temporal trends. All visualizations were created using R.

### 2.7 Dynamics Clustering and Ontology Mapping

Naïve clustering of temporal phosphodynamics was performed by K‐means nearest neighbor set to parse 42 groups using SciPy [26]. Resulting clusters were assessed for gene ontology enrichment via GO, and enriched nodes were coupled together for heatmap generation. Seaborn clustermap was used for correlation‐enriched, hierarchical dendrogram mapping to enhance ordering of heatmaps [27].

### 2.8 Time‐Radiation Network Maps

Network maps were generated in Cytoscape using StringApp [28]. Phosphoproteomic quantitation was then superimposed as concentric circles where time radiates from the central node outward for each network member present in the normalized phosphoproteomic nigericin time‐course dataset.

### 2.9 ASC Speck Quantification

5 × 10^4^ U937 cells were plated in 50 µL of 5% HI‐FBS RPMI + 2 mM Glutamine + 20 ng/mL PMA per well of a 384‐well imaging plate. Cells were allowed to differentiate for 1 day prior to administering an additional 50 µL of 400 nM KDO‐Lipid A (200 nM final concentration) for 18 h, where indicated for priming. Primed cells were then stimulated with 10 µL of 100 µM nigericin (10 µM final) for the indicated times. Cells were simultaneously fixed in 1.6% PFA for 10 min, followed by solubilization in 70% ethanol for at least 5 min. Cells were rehydrated in PBS and were blocked in 2% BSA‐PBS for 15 min, followed by staining with 1:1000 ASC (TMS1) [EPR10403] antibody (Abcam) for 1 h in blocking buffer. Wells were then stained with 2 µg/mL HST 33342 in blocking buffer with 1:2000 Alexa 647 Dk anti‐Rb secondary (30 µL total). Cells were washed 2× with PBS and were imaged on a CX7 high content imager using spot‐detection on a fixed threshold to detect ASC specks.

### 2.10 Draq7 Imaging

5 × 10^4^ U937 cells were plated in 50 µL of 5% HI‐FBS RPMI + 2 mM Glutamine + 20 ng/mL PMA per well of a 384‐well imaging plate. Cells were allowed to differentiate for 1 day prior to administering an additional 50 µL of 400 µM KDO‐Lipid A (200 µM final concentration) for 18 h, where indicated for priming. Primed cells were then stimulated with 10 µL of 100 µM nigericin (10 µM final) for the indicated times. Cells were stained with 2 µg/mL HST 33342 and 1:300 Draq7 for 15 min and were imaged within an environment control chamber on a CX7 high content imager using laser autofocus and light intensities set at 10% intensity with 3 gain to minimize phototoxicity. Images were acquired every 10 min for 6 h in an environmental control chamber (60% humidity:5% CO_2_) and were analyzed using a fixed threshold with no object detection to assess augmented Draq7 intensity over background fluorescence.

### 2.11 Capillary Gel Electrophoresis (CGE)/Western Blotting

CGE was performed on the JESS instrument according to manufacturer protocols. 4 × 10^6^ U937 cells were differentiated with PMA for 2 days before priming with 200 KLA for 18 h where indicated. Culture medium on cells was changed to Optimem and 10 µM nigericin was used to trigger the cells for 1 and 3 h. Cells were chilled on ice for 10 min followed by scraping and pelleting. Supernatant was then precipitated in 80% Acetone (final concentration). Cells were lysed in RIPA buffer (0.02% NP40, 0.002% SDS, Protease inhibitor cocktail [2×], Roche PhosStop [2×]) for 10 min. Insoluble material was pelleted at 10,000 × *g* for 10 min and extract was transferred to 5× LDS sample buffer. Briefly, cell lysates a precipitated supernatants were mixed 1:1 for ratiometric analysis of lysate and secreted proteins. Samples were heated at 70°C for 10 min before running CGE with 1:100 goat anti‐IL‐1b and 1:100 rabbit anti‐actin with respective 647 and IR fluorescent secondaries.

## 3 Results

**FIGURE 1:**
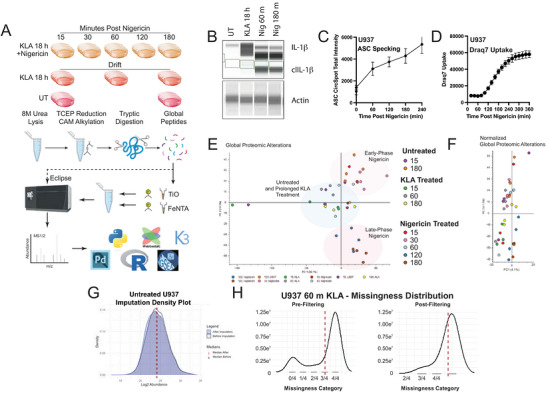
(A) Experimental design focused on assessing proteomic and phosphoproteomic changes following nigericin triggering in 18 h KDO‐Lipid A (KLA) primed, PMA differentiated U937 macrophages. Samples were designed in drift replicates to assess batch effects and parallel drift due to account for concurrent signaling. Cell pellets were washed and lysed in 8 M urea, protein was quantitated, and samples were processed via standardized peptide generation via trypsin. Peptides were quantitated following graphite enrichment and were split between global proteomic analysis and phospho‐peptide enrichment on sequential TiO2 and Fe‐NTA. (B) Capillary gel electrophoresis of IL‐1β induction by KLA and cleavage in response to nigericin as validation of prime and trigger stimuli. (C) ASC speck immunofluorescent imaging quantitation for indicated treatment conditions and times to gauge correlative alterations with inflammasome formation. (D) Cell lysis assessment by Draq7 uptake to gauge correlative pathway kinetics to the onset of pyroptotic cell lysis. (E) PCA of proteins detected across samples run sequentially on an Orbitrap Fusion Eclipse spectrometer. Colored ovals show untreated and KLA treated samples (blue) and those treated with nigericin (pink). (F) Corresponding PCA axes from 1E following protein normalization and exclusion of 1 outlier sample reduced variability by ∼1 log. (G) Protein distribution plot comparing raw (black line) and normalized (red line) sample values for untreated (0 min). (H) Missing‐ness calculation for proteins s over sample replicated following a 2/4 representation cutoff prior to performing regressive imputation.

To examine global proteomic alterations in U937 macrophages exposed to the inflammasome trigger nigericin across a 180‐min time course, we performed unlabeled shotgun proteomics of four independently processed biological replicates to verify statistical representation and repeatability of technical processing across samples (Figure 1A). We included KLA‐primed, but non‐triggered, parallel drift samples to account for ongoing proteomic alterations emanating from the differentiation or priming steps due to the sequential nature of inflammasome checkpoint progression (Figure 1A). We also included an additional untreated drift control for further batch normalization as an additional check for spectrometer performance (Figure 1A). As traditional short‐term cellular models of inflammasome activation can exhibit overlapping signaling from recent cell priming, we employed a previously established extended differentiation and prime regimen in U937 cells that permits prime‐dependent signaling to return to baseline and better mimics kinetics observed in primary human monocyte‐derived macrophages (hMDM) [17]. We first validated that priming and subsequent inflammasome activation following nigericin treatment led to the expected upregulation and cleavage of IL‐1β cytokine (Figure 1B). We further validated the kinetics of inflammasome assembly with ASC‐speck high‐content imaging, while the onset of pyroptosis and cell permeation was evaluated by a live‐cell Draq7 uptake (Figures 1C,D). Here, a significant amount of ASC specking occurs within 1 h of nigericin stimulation, while Draq7 uptake follows at around the 2‐h mark (Figure 1C,D). This segmented temporality allowed us to select early timepoints between administration of nigericin and downstream ASC specking as well as later‐phase timepoints occurring between inflammasome formation and pyroptotic cell lysis (Figure 1A).

This treatment regimen was scaled to generate urea lysates from cell pellets for standardized TCEP reduction, chloroacetamide alkylation, and trypsinization. Peptides were then split to perform global proteomic analysis as well as undergo TiO_2_ and Fe‐NTA column enrichment for phosphoproteomics (Figure 1E). Phospho‐enriched samples were injected into an Orbitrap Fusion in DDA mode to enhance reliability of attained MS2 spectra. We then assessed variation in the global peptide distribution by PCA analysis (Figure 1F). Although variance in shotgun proteomics is common, we sought to reduce distribution variance by normalizing peptide scores. After removal of one outlier, normalization reduced the major axis of distribution variance by a full log, driving PC1 from 30.1% variance to 4.1% (Figure 1G). We then verified that normalization had little impact on the overall peptide distribution with each sample set (Figure 1H). Further exclusion of missing peptide quantitation within our replicates were performed if detection failed in 2 or more of the 4 samples while interpolation was used to fill remaining missing peptide quantitation (Figures 1I, S1A,B).

**FIGURE 2:**
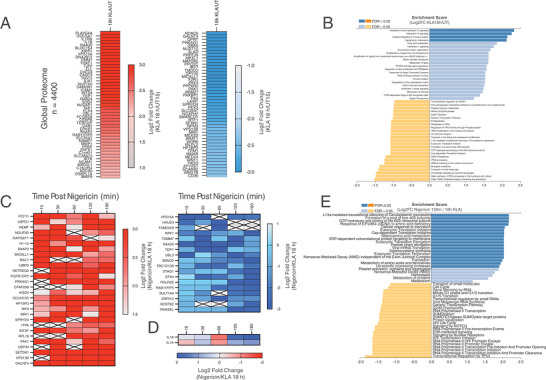
(A) Out of the 4400 proteins quantified in the global proteome, heat maps depict the top and bottom 50 proteins enriched (red) and depleted (blue) in response to 18 h of 200 nM KLA treatment. (B) Gene ontology calculation for enriched pathways in top and bottom differentially expressed proteins following 18 h 200 nM KLA treatment. (C) Top and bottom (cutoff −/+ 1.0 Log2FC) differentially perturbed proteins following indicated timings of 10 µM nigericin treatment of 18 h 200 nM KLA primed U937 macrophages. (D) Gene ontology calculation for enriched pathways in top and bottom differentially expressed proteins 120 min after 10 µM nigericin treatment of 18 h 200 nM KLA primed U937 macrophages.

Using the cleaned dataset, we first assessed priming‐induced alterations within the global proteome following 18 h KLA administration. Here we find that out of 4574 proteins detected, 85 (1.85%) were upregulated beyond a log‐2‐fold change of 1.5, whereas 124 proteins (2.71%) were downregulated. Among the most highly enriched proteins are well‐known prime‐induced pro‐IL‐1β and IL‐1RN (Figure 2A). Similarly, the top ontological enrichments from the global proteome following extended KLA priming were the well‐established IL‐4/13, IL‐10, and IL‐1 signaling networks (Figure 2B). Curiously, pro‐apoptotic and anti‐inflammatory regulators FAS and NLRX1 are amongst the most heavily depleted proteins during extended priming (Figure 2A). Additional depletions in Protein Kinase A, PAK1, and MAP2K5, which subsume non‐traditional roles in refining inflammatory signaling in monocytes, could suggest cultivation of a hyperinflammatory and apoptosis‐refractory base‐state (Figure 2A).

**FIGURE 3:**
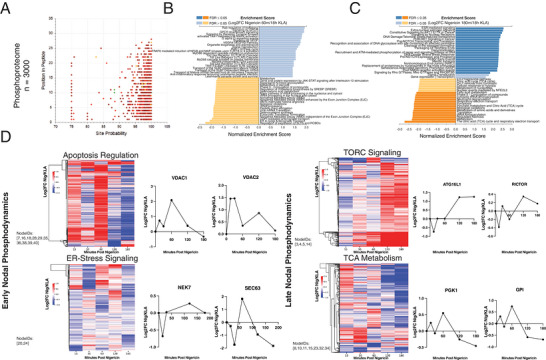
(A) Site identification confidence distribution of phosphorylated peptides from the 3000 phosphoproteins identified. (B and C) Gene ontology calculation for enriched pathways in top and bottom differentially abundant phosphoproteins 30 (B) and 120 min (C) after 10 µM nigericin treatment of 18 h 200 nM KLA primed U937 macrophages. (D) Heatmaps of phosphoprotein dynamics partitioned by K‐means clustering assessed after nodal merger by ontological enrichment corresponding to 3B. Enriched: Red. Depleted: Blue. Left: Ontologies impacted within 1 h of nigericin treatment. Right: Ontologies impacted after 1 h of nigericin treatment. Merged K‐node identifications are depicted in brackets for each heat map. Heat maps are accompanied by kinetic graphs of representative members of associated ontologies.

We then investigated global proteomic alterations in response to the nigericin trigger over time. We find that few proteins are enriched or depleted above a 1.5 log‐2 fold‐change threshold across this time‐course showing 28 proteins enriched and 18 proteins depleted (0.6% and 0.4%, respectively) (Figure 2C). Far more prevalent within the nigericin time‐course are small, but statistically significant fluctuations such as those seen in inflammatory cytokines IL‐1α and IL‐18 which show depletion after they are cleaved and released following inflammasome activation (Figure 2D). These small alterations result in ontological enrichment of translation components and depletion in factors involved in stress‐induced transcriptional repression following 120 min of nigericin treatment (Figure 2E). This co‐occurrence supports prior observations that unlike apoptotic quiescence, inflammasome activation, and pyroptosis represent a more active and directed inflammatory response, that is, supported by transcription and translation. We then applied the same normalization steps to the dataset arising from the phosphoproteomic mass spectrometry runs (Figure S1C,D). Of the phosphopeptides identified (Table S3), we were able to achieve nearly 80% site identification at a probability of >90% suggesting robust enrichment from our IMAC pipeline and high sensitivity through MS2 splitting (Figure 3A). Ontologic mapping further revealed protein phosphorylation associated with the canonical glycolytic and TCA cycles, prominent members of MAPK signaling cascades, and apoptosis regulating proteins was enriched after 30 min of nigericin administration (Figure 3B). In tandem, proteins associated with translational control and oxidative stress response pathways were phosphodepleted (Figure 3B). Consistent with the notion of multiphasic signaling responses downstream of nigericin treatment, we find that acute response pathways harboring phosphoenrichment were very different from the phosphoenriched peptides after 120 min of nigericin treatment. At these later timepoints, we observe prominent protein dephosphorylation associated with the TCA cycle and lipid metabolism and concurrent phosphorylation in estrogen receptor signaling, AKT/TOR signaling, and DNA damage response pathways (Figure 3C).

To better address the ontologic phosphoprotein alterations that correlate over time, we used K‐means clustering to assign phosphoprotein quantifications into 42 distinct groups. Since this method clusters only correlated signaling dynamics, we then used ontology enrichment to couple nodes into ontologically enriched subsets where both correlative and anti‐correlative enrichments can be visualized via a heat‐map (Figure 3D, Table S4). This allowed us to clearly display phase‐enriched phosphoprotein alterations in signaling cascades over time.

Here, we show early phasic phosphorylation enrichment of apoptotic regulatory proteins and associated VDAC mitochondrial channel subunits spiking at 15 and 60 min before diminishing past 120 min after nigericin treatment (Figure 3D), a finding consistent with prior reports indicating VDAC involvement prior to inflammasome formation [29]. Intriguingly, a less clear pattern exists within ER‐stress‐associated nodes where oscillatory patterns appear less cohesive within the ontology and appear more subset‐distinct. Notably, microtubule regulatory proteins, including the NLRP3‐inflammasome‐related NEK family (3/6/7) [30, 31], are found within this group and show rapid depletion of phosphorylation before recovering at 60 min (Figure 3D). These observations are largely consistent with the differential impact of NEK7 phosphorylation and related IKKβ conditional NEK7 bypass in inflammasome seeding responses [32, 33].

In stark contrast to ontologies harboring strong acute phospho‐enrichment, we find that TORC enrichment generally augments over the nigericin time‐course with maxima following 120 min of treatment (Figure 3D). This timing is particularly intriguing as recent reports suggest involvement of TORC2 in cell‐death inhibition in response to DNA‐damage [34, 35]. In stark contrast, we see a robust depletion of phosphorylation in TCA‐related proteins after 120 min of nigericin treatment (Figure 3D). The timing of phosphorylation flux in this ontology may relate to rapid, nigericin‐mediated depolarization of the mitochondria. This in turn promotes metabolic redirection from TCA cycle‐metabolism to compensate for the loss of proton gradient necessary for oxidative phosphorylation [36], while marked dephosphorylation at later timepoints align with TCA shutoff during inflammasome activation [37]. These findings could support the existence of a phasic transition from compensatory stress cascades into terminal cell death commitment programs that occur alongside the inflammasome assembly and pyroptotic cell death.

**FIGURE 4:**
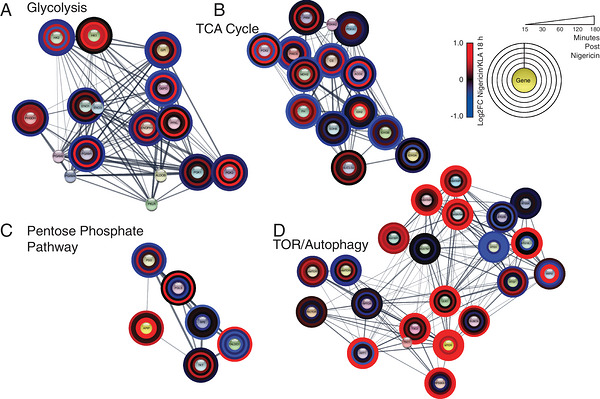
(A–D) Time radiation network maps of nigericin‐induced phosphorylation of canonical members of glycolysis (A), the pentose phosphate pathway (B), the TCA cycle (C), and TOR/Autophagy signaling (D) corresponding to findings in Figure 3D.

We next focused on phosphorylation within the core components of energy supply pathways by generating time‐radiation network maps. Here, many core enzymes in the glycolytic and TCA cycles display trends where phosphorylation is observed within 60 min of nigericin treatment, followed by a prominent depletion past 120 min (Figures 3D and 4A,B). This is clearly evident for hexokinase‐1 and PHGDH within glycolysis (Figure 4A) and IDH2, SDHB, FH, SUCLG2, and MDH2 in the TCA cycle (Figure 4B). These alterations are consistent with other reports indicating drastic alterations within the TCA cycle [38, 39], ranging from electron transport chain dysfunction and utilization of succinate between complex II (SDHB) and complex III preceding fumarate hydratase (FH), which we find are respectively depleted and heavily augmented for phosphorylation (Figure 4B). Similarly, reports indicating IDH plays a role in α‐ketoglutarate accumulation during glutaminolysis [40, 41] may explain a very early augmented phosphorylation of this enzyme. The observation that both the glycolytic cascade and the TCA cycle robustly deplete their phosphorylation state after 120 min of nigericin suggests a major shift from traditional energy sourcing, consistent with recent reports showing gross mitochondrial dysfunction after nigericin treatment [37]. Similarly, we further observe alterations in the pentose phosphate pathway, indicating widespread metabolic reprogramming (Figure 4C). Consistent with recent reports of active apoptosis inhibition following stimulation with inflammasome triggers [42], we also observe a prominent phosphorylation of APIP that serves dual roles in the methionine salvage pathway as well as acting as a potent AKT:ERK‐driven APAF1:Caspase 9 inhibitor to prevent intrinsic apoptosis (Figure 4C) [43].

In contrast, phosphorylation flux among components involved in canonical metabolism is quite different to that observed for mTOR/AKT signaling cascades, where many core components exhibit delayed phosphorylation increases following nigericin treatment (Figure 4D). This suggests that cellular recycling cascades normally invoked by mTOR inhibition, via dephosphorylation, are limited as cells commit to pyroptosis. We do observe phosphorylation of the autophagy adaptor TAX1BP1, autophagophore closure protein ATG16L1, and GABARAP family LC3 homologs, suggesting possible involvement of autophagic processes distinct from traditional starvation‐induced autophagy (Figure 4D) [44, 45]. Interestingly, TORC2 is additionally implicated in the metabolic shift out of oxidative phosphorylation and into fermentation via the AKT1 signaling axis further highlighting likely involvement in metabolic perturbations in this response [46, 47, 48]. Temporality of LAMTOR phosphorylation suggest phasic regulation of the Ragulator complex, that is, augmented for 60 min after nigericin treatment, followed by dephosphorylation of LAMTOR and phosphorylation of RICTOR and RPTOR, selective components of TORC2 and TORC1 respectively (Figure 4D). These phosphoprotein fluctuations may play roles in refining metabolic signaling axes utilized early versus late following nigericin stimulation, and they are largely consistent with reported roles for RagA and TORC in late‐phase activation of the pore‐forming protein GSDMD [49]. Together, our observations of dynamic phosphoprotein flux among core metabolic pathways suggests that cellular metabolism plays a central regulatory role in the inflammasome trigger response.

**FIGURE 5:**
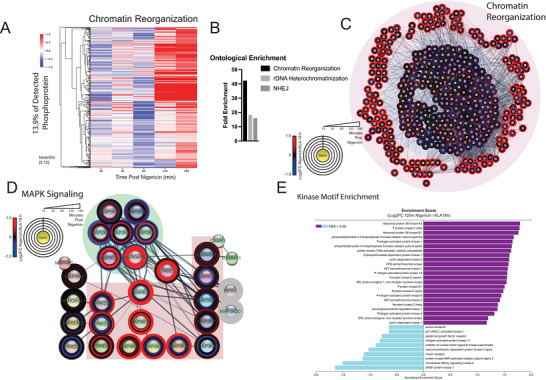
(A and B) Dynamic heatmaps of chromatin‐remodeling protein phosphorylation over time following nigericin treatment (A) and respective ontological enrichment (B). (C) Time radiation network map of phosphorylation dynamics within chromatin‐remodeling proteins. Underlay of blue indicates 47% of phosphoproteins identified with a log2FC < 1 while underlay of red indicates a log2FC > 1 at 180 min after nigericin treatment compared to overnight primed cells. (D) Time radiation network map of phosphorylation dynamics within core or related to MAPK‐associated signaling kinase cascades. Underlay of green indicates phosphoproteins identified that cluster with TCA and metabolism nodes while red underlay indicates phosphoproteins clustering with chromatin condensation nodes. (E) Phospho‐motif enrichment analysis for the human kinome depicts broad utilization of various MAP‐kinase branches following nigericin treatment.

Although metabolism and TOR signaling nodes represent 10.0% of the total phosphoproteins identified in our dataset, chromatin restructuring represents 13.9% of significantly altered protein phosphorylation following nigericin treatment, and nearly all members exhibit phosphoenrichment at the later 120 and 180 min timepoints after nigericin treatment (Figure 5A). These proteins are ontologically enriched in chromatin restructuring pathways as well as those responding to DNA fragmentation (Figure 5B). Interestingly, structural proteins participating in DNA binding as well as mobilization and reorganization of chromatin tend to have fewer phosphopeptides detected (Figure 5C—inner blue underlay) while those that are thought to play dynamic organizational roles tend to be more strongly phosphorylated and often display activation around the 120‐min timepoint (Figure 5C—outer red underlay). This strongly supports prior evidence of a caspase 1‐driven DNA fragmentation distinct from nuclear disassembly during the apoptotic pyknosis:karyorrhexis cascade [50].

To better understand temporal utilization of predominantly bimodal MAPK signaling responses, we examined activation kinetics of core MAPK signaling and related immune kinases and associated substrates (Figure 5D). We find that ERK3/6 (MAPK4/6) and p38a (MAPK14) signaling axes respond early following nigericin treatment consistent with observed co‐clustering in metabolic signaling nodes (Figures 5D and 3D). Tangentially, we find prominent late‐phase responses for PKCβ/δ, RIPK1/3, and MAP3K5/6/7 (Figure 5D) consistent with prior functional reports [17, 51, 52, 53]. These data are further supported by a large degree of phosphorylated substrate enrichment for MAPK14 (p38α), MAP3K5 (ASK1), MAPK9 (JNK2), and several PKC family members following 120 min of nigericin treatment (Figure 5E). These data together provide strong evidence of phasic MAPK and PKC signaling responses during the inflammasome trigger step and further suggest which signaling cascades influence temporally distinct cellular alterations associated with inflammasome formation and the downstream pyroptotic processes associated with lytic inflammatory cell death.

## 4 Discussion

Although phosphodynamics downstream of specific toll signaling has been heavily studied [6, 8, 9, 10, 54], we now extend our understanding to post‐translational modifications within the cellular inflammatory response that follows an inflammasome trigger. To complement other studies focused on specific phosphorylation of core inflammasome proteins [15, 18, 19, 20, 21, 22, 23, 55], we utilized a whole phosphoproteome approach to get an unbiased snapshot of parallel signaling cascades invoked by the inflammasome trigger nigericin.

We demonstrate here that technical and statistical advances in shotgun phosphoproteomics can facilitate broad examination of phosphoproteome dynamics associated with inflammasome triggers. Although all mass spectrometry studies are influenced by sample extraction and preparation methodologies, missingness and representation variability can be partially overcome by increasing the number of biological replicates receiving parallel processing, including rational exclusion criteria, and interpolating missing values within the final dataset. Even with sufficient detection, differences in sample ionization within a complex mixture can vary, an issue we overcame by inter‐replicate normalization. Under our conditions, these adjustments limit technical variation across experimental samples and do not affect the detected peptide distribution, supporting the validity of downstream relative quantitation (Figure 1). Although no conclusion can be made for proteins absent following this process, the resulting peptide quantifications facilitate unbiased time‐dynamic analyses in response to stimulatory agonists.

Within our dataset, we clearly observe expected enrichment of IL‐1 cytokines in the global proteome following KLA treatment while nigericin treatment minimally impacts total protein levels (Figure 2). This low global protein variance allows direct comparison of omic‐level phosphopeptide abundance without additional ratiometric normalization to protein load. Using this approach for our phosphoproteomic dataset highlighted known kinase responses such as a well‐characterized nigericin‐induced phosphorylation of p38α (MAPK14), as well as TOR/AKT phosphorylation, that is, thought to be essential for pyroptotic pore formation (Figures 3 and 4) [17, 49, 56]. Ontology mapping following nigericin treatment revealed categorical shifts in dynamically leveraged pathways preceding pyroptotic lysis. Further K‐means clustering and iterative ontological coalescence clustered correlated and anticorrelated phosphorylative fluxes. These associated dynamics can be used to assess signaling through multi‐member cascade inference, and although the associative strength of congruent time‐correlative association is augmented, it is not a causal assessment and must be interpreted with care. We also note that we cannot assume that phosphorylation is necessarily an activating cue since dephosphorylation and kinase inactivity is known to activate several stress pathways such as autophagy [20, 57]. Furthermore, interpretations for proteins such as VDAC1 and VDAC2 that undergo site‐specific phosphorylation that can either augment or inhibit their association with hexokinases to impact metabolic shifting and cell‐death functions should be interpreted with care (Figure 3) [58]. Though imperfect, ontologically clustered dynamics can be used to inform further causal experiments for specified phenotypic output.

In this study, we find that phosphoregulation within the TOR signaling axis and TCA metabolism trend toward respective enrichment and depletion by 180 min of nigericin treatment (Figures 3 and 4). By examining phosphorylation across the canonical metabolic cascades, we see a peak of phosphoenrichment at 60 min of nigericin, followed by a drastic phosphodepletion across all members, except a few in the pentose phosphate pathway (Figure 4). This coordinated response reflects metabolic findings from a recent report where nigericin causes numerous mitochondrial defects that affect the TCA cycle [37, 59]. The O'Keefe study found that inflammasome agonists inactivate ERK1/2 through mitochondrial stress‐induced DUSP6 activation, leading to the dephosphorylation and activation of TTP (ZFP36). We observed a decrease in MAPK1 (ERK2) phosphorylation at 15 and 30 min after nigericin treatment, followed by recovery at 120–180 min. ZFP36 followed a similar temporal pattern, indicating its regulation downstream of ERK, supporting the existence of a post‐transcriptional checkpoint in inflammasome signaling (Figure S2, Table S1).

Similarly, our apoptosis‐regulatory enrichment is also in line with the reported mechanism preventing caspase 9 activity [43]. Interestingly, this metabolic occurrence does not appear to lead to TOR inhibition, where core TOR signaling members are robustly active throughout nigericin treatment (Figures 3 and 4). These findings could support the role of TOR signaling outside of starvation‐induced autophagy, consistent with a role at the pyroptotic pore [49].

Our data also support a strong role for directed kinase regulation within the chromatin remodeling response associated with lytic pyroptosis [60, 61]. Since we observe phosphorylation of 475 proteins associated with DNA repair, damage, and chromatin remodeling aligned with the onset of pyroptotic lysis following 120 min of nigericin treatment (Figures 1D and 5), we believe it is very likely that DNA‐damage‐induced chromosomal restructuring is a very large, complicated, and directed cellular response to lytic death cascades. In combination with early phosphomodifications in apoptosis‐regulating components (Figures 2A and 4C), we believe these alterations provide an avenue for investigating communication between, and directed selection of, distinct cell death effector arms represented by apoptosis, pyroptosis, ferroptosis, and necrosis, amongst others. Similarly, matching dynamics from MAP3K5/6/7, JNK, p38, RIPK, and PKCβ/δ temporally associate these events from distinct, novel ERK3/6 signaling dominating the onset of nigericin treatment (Figure 5). This dataset provides an unbiased window into phosphoregulatory cascades invoked by nigericin treatment. Delineating which of these signaling components are critical for the progression and regulation of distinct inflammasome formation and pyroptotic cell death events will unlock new therapeutic avenues for the treatment of inflammatory diseases.

## Author Contributions

Conceptualization: C.J.B. and V.B. Methodology: C.J.B., V.B., S.S., and S.H.Y. Software: C.J.B. and V.B. Formal Analysis: V.B., S.S., and C.J.B. Investigation: C.J.B. and V.B. Resources: A.N.L., and I.D.C.F. Data Curation: V.B., S.S., and C.J.B. Writing—Original Draft: C.J.B., I.D.C.F., and A.N.L. Writing—Review & Editing: C.J.B., I.D.C.F., V.B., and A.N.L. Visualization: C.J.B., S.S., and V.B. Supervision: A.N.L. and I.D.C.F. Project Administration: C.J.B., V.B., I.D.C.F., and A.N.L. Funding Acquisition: A.N.L. and I.D.C.F.

## Conflicts of Interest

The authors declare no conflicts of interest.

## Supporting information




**Supporting Figure 1**: pmic70030‐sup‐0001‐FigureS1.docx.

**Supporting Figure 2**: pmic70030‐sup‐0002‐FigureS2.tiff.

**Supporting Table 1**: pmic70030‐sup‐0003‐TableS1.xlsx.

**Supporting Table 2**: pmic70030‐sup‐0004‐TableS2.xlsx.

**Supporting Table 3**: pmic70030‐sup‐0004‐TableS3.xlsx.

**Supporting Table 4**: pmic70030‐sup‐0004‐TableS4.xlsx.

## Data Availability

The datasets were deposited in the ProteomeXchange via PRIDE partner repository [62] with the dataset identifier PXD062818 (phosphoproteome) and PXD062943 (proteome).
