# Supplementary material for: Nigericin‐Triggered Phosphodynamics in Inflammasome Formation and Pyroptosis
Source: Proteomics. 2025 Sep 2;26(8):29–40. doi: 10.1002/pmic.70030 (PMC12673643; doi:10.1002/pmic.70030)
Supplement: Supplementary file 1 — Supporting Figure 1: pmic70030‐sup‐0001‐FigureS1.docx. [file PMIC-26--s005.docx]

**Figure S1:**

1. PCA of normalized global proteins detection across samples run sequentially on an Orbitrap Fusion Eclipse spectrometer. All samples cluster together except 1 outlier suggesting high fidelity of relative peptide quantitation in each sample.
2. Heat map representation of average (N=4) normalized global protein intensities in each condition.
3. PCA of normalized phosphoproteins detection across samples run sequentially on an Orbitrap Fusion Eclipse spectrometer to assess variability between sample runs.
4. Heat map representation of quantified normalized phosphoprotein intensities for each sample quadruplicate.
