# Supplementary figures and images for: Nigericin‐Triggered Phosphodynamics in Inflammasome Formation and Pyroptosis
Source: Proteomics. 2025 Sep 2;26(8):29–40. doi: 10.1002/pmic.70030 (PMC12673643; doi:10.1002/pmic.70030)

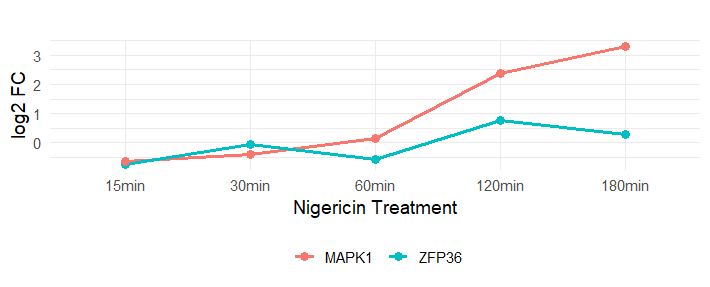

Supplement: Supplementary file 2 — Supporting Figure 2: pmic70030‐sup‐0002‐FigureS2.tiff. [file PMIC-26--s002.tiff]
